# Techno-overload and employee outcomes: relationships with job insecurity, turnover intention, and occupation insecurity among Lithuanian employees

**DOI:** 10.3389/fsoc.2026.1782891

**Published:** 2026-07-27

**Authors:** Laima Okunevičiūtė Neverauskienė, Dalia Bagdžiūnienė

**Affiliations:** 1 Lithuanian Centre for Social Sciences, Vilnius, Lithuania; 2 Institute of Psychology, Vilnius University, Vilnius, Lithuania

**Keywords:** digital technologies, techno-overload, job insecurity, turnover intention, occupation insecurity, employees

## Abstract

**Introduction:**

Information and communication technologies are rapidly integrating into everyday life and work processes. Nowadays, without them, innovations, human development, and business growth are impossible. At the same time, this integration increases the likelihood of negative consequences for employees, particularly technostress and techno-overload, as one of its creators.

**The aim:**

Based on the Job Demands–Resources Theory and the Social Exchange Theory, this research examines the role of techno-overload in relation to employee job insecurity, turnover intention, and occupation insecurity among Lithuanian employees.

**Method:**

The cross-sectional study sample involved 754 employees from state and private organizations, with 54.8% female representation. The average age of participants was 41.9 years (*SD* = 12.06). The survey was conducted using Computer-Assisted Telephone and Web Interviewing. Descriptive statistics, correlation analyses, and structural equation modeling were applied for data analysis.

**Results and conclusion:**

Techno-overload was positively associated with employees' job and occupation insecurity. Job insecurity mediated the relationships between techno-overload and both turnover intention and occupation insecurity. The results complement research on the links between techno-overload and employee insecurity in the labor market, reveal the role of job insecurity in the relationships between techno-overload and employee outcomes, and the value of more comprehensive studies of occupation insecurity—an important construct in employment research. Research limitations, theoretical and practical implications are discussed.

## 1 Introduction

Digitalization, defined as the widespread adoption of digital information and communication technologies, is a new reality and an evolutionary force transforming the modern world of work (Colbert et al., 2016). The adoption of digital technologies represents a fundamental business strategy of automating work processes, creating a new digital work environment, providing collaboration, communication, and knowledge management tools that accelerate work processes and aim to increase operational efficiency, employee engagement, and flexibility (Khashkhashneh et al., 2019; Williams and Schubert, 2018).

The survey on the Impact of Algorithmic Management and Artificial Intelligence in the Workplace, conducted in 27 EU member states, revealed that a majority of EU workers (over 90%) use digital devices, tools, and equipment to do their jobs (Gonzalez Vazquez et al., 2025; p. 35). Lithuania is also on this path, implementing this transformation, prioritizing the digitalization of the workplace in its national strategic agenda, and considering it a key driver of economic growth, competitiveness, and societal resilience. According to data from the State Data Agency of Lithuania, in 2023, approximately 83.2% of working persons used information technologies every day (State Data Agency, 2023). In 2025, this number increased to 94.0% (https://osp.stat.gov.lt/statistiniu-rodikliu-analize?hash=206a1641-d322-40ef-8bb7-4fb5d63e27de#/). The importance of digital sovereignty, as an issue for the country's future existence in the context of global changes, is emphasized in the State's Strategic Document Lithuania's Vision for the Future Lithuania 2050 (Seimas of the Republic of Lithuania, 2024). As stated in this document, automation and digitalization have the potential to significantly enhance business productivity and open new market opportunities, while also noting that “automation will also likely pose a significant threat to the category of routine tasks and medium-skilled workers as about 63% of jobs in Lithuania could be automated in the future” (p. 24). Therefore, Lithuania represents a meaningful setting for investigating how technology-related work requirements are associated with employees' employment-related perceptions and behavioral intentions.

Widespread adoption of advanced digital technologies may not only reduce the number of jobs. In this environment, employees face pressure to learn and quickly apply these technologies, leading to an increased variety of responsibilities: managing workload and speed, while simultaneously strengthening professional abilities and developing new ones. They must process larger amounts of information or manage multiple digital tasks simultaneously. Research indicates that technology-related job requirements do not act as additional demands; they are directly involved in work processes and partially correct them. As Tarafdar et al. (2024) state, technological load pushes employees beyond their comfort zone by encouraging them to constantly learn, acquire new skills, and expand their professional horizons. However, the positive changes diminish as these demands approach the optimal work capacity. The reaction to technology-related job demands is similar to a threshold: even a slight increase in volume and complexity can trigger negative reactions and exhaustion. Karr-Wisniewski and Lu (2010) also note, “...increased usage of technology tools does not always lead to increased work productivity; rather, sometimes it actually can be counterproductive” (p. 1061).

Kim and Kim (2024) note that technological transitions may impose psychological costs even when economically beneficial, possibly reflecting increased work intensity, difficulties in adaptation, or concerns about future job security despite current improvements. One negative outcome is technostress, the stress experienced by individuals who use information and communication technologies in the workplace (Ragu-Nathan et al., 2008). Among the creators of technostress, techno-overload has become particularly relevant. It occurs when technology increases the pace and volume of work, forcing employees to work faster and handle excessive amounts of information and communication. As organizations continue to digitalize their work processes, employees may experience increasing pressure to manage a variety of technological tools and meet accelerating work demands. Research reveals that techno-overload may create undesirable outcomes for employees and the organization as a whole, including high cognitive or emotional load, exhaustion, low job satisfaction and performance, disengagement at work and from citizenship or innovative behaviors (Tarafdar et al., 2007; Ragu-Nathan et al., 2008; Tarafdar et al., 2019; Salanova et al., 2013; Chandra et al., 2019; Borle et al., 2021; Ingusci et al., 2021). In the long term, constant technological pressure can be linked to employees' perceptions of professional competence and job stability. Those who find it difficult to cope may fear losing their jobs or becoming less valuable, feel insecure in their current position, or even in the external labor market. In addition, prolonged technology overload can impair job satisfaction and organizational commitment, thereby increasing turnover intentions and, in some cases, actual turnover.

In this context, research examining the links between techno-overload and employees' relationships with the organization and profession is relevant. There remains a lack of research examining its relationships with potential employment-related outcomes: employee feelings of security/insecurity in their current job position and two more distal potential outcomes—intention to leave the organization as the attitude toward terminating employment in the concrete organization, and the perception of possible occupation insecurity in the future due to technological changes. For this study, we selected the technology overload outcomes based on several assumptions. When it comes to the continuity of work within a particular organization and, more broadly, of a professional career, three employment-related aspects can be distinguished: job insecurity, intention to leave the organization, and occupation insecurity. Lithuania is analytically and theoretically relevant for examining the consequences of technology overload as it reflects broader labor market developments related to digitalization and technological change. Today, technology is not only an additional source of workload; it can also change workers' perceptions of their future employability and professional stability. Examining whether technology overload contributes to employment-related insecurity, including concerns about current job continuity and the future occupational viability of Lithuanian employees, can enrich research in this area and therefore contribute to a deeper understanding of how technological transformation shapes workers' perceptions of insecurity and work-related outcomes.

Job insecurity in general refers to employees' concerns about their work-related future within the current organization (De Witte et al., 2010), and more specifically, about the possibility, when implementing changes, to keep the job (quantitative job insecurity) or valued job characteristics (qualitative job insecurity) (Vander Elst et al., 2014).

Another potential consequence examined was employee turnover intention. Voluntary turnover is defined as an employee's active, voluntary decision to leave an organization (Tett and Meyer, 1993). It is analyzed as a form of dysfunctional withdrawal behavior for the organization (Carlson et al., 2017). and is the best predictor of actual turnover (Hom et al., 2017). The search, selection, social, and professional adaptation of new employees takes time and requires costs. Of course, the intention to leave does not necessarily mean the employee has already decided to leave or is actively seeking another job. The decision to stay and continue working despite increasing technological requirements may be related to a wider range of micro-level individual, mezzo-level organizational, and macro-level labor-market factors. A person may be satisfied with financial rewards, organizational welfare programs, or a positive psychological work environment, but lack confidence in their professional or job-seeking competencies, while in the external labor market, choices may be limited by a low supply of jobs or unsatisfactory working conditions offered by other organizations (Bhat et al., 2023). However, this intention is important because it signals an imbalance between the organization's evolving job requirements and the internal resources needed to fulfill them. In the long run, turnover intention may become a subtle yet persistent inner disturbance, negatively associated with feelings of wellbeing, work engagement, job satisfaction, and work motivation (Bolt et al., 2022).

Occupation insecurity refers to the anxiety and uncertainty employees experience about significant future changes in their profession resulting from technological developments in the labor market (Roll et al., 2023; 2025). The technology overload initially manifests as more intense work demands, driven not only by the volume of tasks but also by changes in their content and complexity. Continued technological acceleration gradually depletes workers' adaptive resources and raises concerns about their ability to remain professionally relevant in increasingly digitalized professions. As a result, the likelihood of continuing a professional career in the same field may decrease.

To conclude, although technological overload is often understood as the intensification of work caused by technology, its consequences can go beyond direct workload pressures. Previous research on technological stress has focused mainly on outcomes such as burnout, emotional exhaustion, work engagement, job satisfaction, and work-life conflict. While these studies reveal a wide range of consequences of technology-related stress, relatively little attention has been paid to employment-related insecurity. In particular, occupation insecurity has received very limited attention in technostress research. This is an important research gap that our study seeks to fill, at least in part. In a highly digitized work environment, workers are constantly adapting to new systems, processing increasing amounts of information, and responding quickly to changing technological demands. The constant pressure can gradually deplete inner adaptive resources and reduce confidence in the ability to remain professionally relevant. As technological demands change rapidly, workers may perceive that their competencies are at risk of becoming obsolete or inadequate to meet new demands, leading to concerns about the long-term viability of their profession.

Drawing on the Job Demands-Resources Theory (Demerouti et al., 2001; Bakker and Demerouti, 2017) and the Social Exchange Theory (Blau, 1964), we examined the links between techno-overload and employee outcomes, including staff retention and future career prospects.

The primary research question was whether and how techno-overload is associated with employees' job insecurity, turnover intention, and occupation insecurity.

## 2 Theoretical framework and literature review

### 2.1 Digital technologies and techno-overload

The rapid digitalization of work processes reshapes the work environment and creates conditions in which employees must constantly learn, master, and apply new technologies; adapt to the pace of work and large volumes of information; and often contend with reduced direct social contact (Kumar, 2024; Pansini et al., 2023). Borle et al. (2021) conducted a systematic review of research in this area, emphasizing that remote work facilitated by mobile devices is often perceived as increasing employee autonomy. Information technologies enable tasks to be performed anywhere and at any time, but there is another side to the coin—the restriction of autonomy and increased external control. Mazmanian et al. (2013) state that a spiral of increasing work involvement gradually develops and “…generates an autonomy paradox—that reliance on mobile devices both increases and diminishes professionals' autonomy” (p. 2). Continuous connection to work, when there is no possibility to disconnect, may increase work involvement but can also diminish autonomy, potentially resulting in negative outcomes and becoming a source of technostress (Abdulkareem et al., 2024). Karr-Wisniewski and Lu (2010) emphasize the non-linear impact of technology-related load on productivity. Although information technology tools are supposed to help employees increase productivity, overload could contribute to productivity loss. The authors describe this relationship as a “performance paradox” (p. 1062). When the amount of new technology or information. Slightly but constantly increases it reaches the worker's acceptance threshold and exceeds their ability to absorb it. A further increase in technological load no longer helps to perform a job; on the contrary, it demotivates and exhausts, becoming a source of stress or burnout. Decataldo and Fiore (2022) also state that “Only recently the focus of research on technologies and the Internet has moved from the issue of ‘use' to that of ‘overload' and the negative consequences of excessive use” (p. 80).

Technostress is defined by Ragu-Nathan et al. (2008) as “…the phenomenon of stress experienced by end users in organizations as a result of their use of information communication technologies” (p. 417). Tarafdar et al. (2007, p. 315) and Ragu-Nathan et al. (2008, p. 425) include techno-overload among the five most important technostress creators, along with techno-invasion, techno-complexity, techno-insecurity, and techno-uncertainty (Tarafdar et al., 2007, p. 315). They explain techno-overload as the increasing pace and workload resulting from the use of technology. Finstad et al. (2024) emphasize that technological overload occurs when the workload associated with technology use must be completed under tight deadlines. Authors of systematic reviews present a wide range of possible sources of technostress (Bahamondes-Rosado et al., 2023; Kumar, 2024; La Torre et al., 2019; Nastjuk et al., 2024). However, it is recognized that technology overload is one of the most significant contributors (La Torre et al., 2019; Borle et al., 2021). Information overload and the need to respond to it necessitate employees to remain connected and accessible at all times (Kumar, 2024). If this communication continues after the working day, it blurs the boundaries between work and personal life (Decataldo and Fiore, 2022). In this way, the integration of technology leads to changes in the work environment and organizational culture, contributing to technostress and, in turn, to negative consequences for employees, such as job dissatisfaction, disengagement from work, and even work-life imbalance (Salanova et al., 2013).

On the other hand, recent empirical studies reveal that overload can be positive, motivating, and engaging. For example, Li and Wang (2021) reported that techno-overload was positively associated with university teachers' work performance. Similarly, Wang and Yao (2023) found that novice teachers tended to appraise techno-overload as a challenge rather than a hindrance. Ren et al. (2025) found that moderate techno-overload stimulates employee digital creativity through digital self-efficacy when employees receive sufficient support from their managers. Hao et al. (2024) argue that in a favorable mastery climate, techno-overload stimulates employee personal growth, work engagement, and performance. Nascimento et al. (2024) demonstrated that those who perceived techno-overload as either challenging or enjoyable were more satisfied with their job and achieved higher productivity.

However, “overload” does not mean “optimal load,” and working under constant technological pressure may be linked to reduced cognitive, emotional, and physical resources, work exhaustion (Gaudioso et al., 2017), negative emotions (Lee, 2016), health complaints (Stadin et al., 2019), insufficient wellbeing (Borle et al., 2021), and reduced productivity (Alam, 2016; Keim et al., 2014).

In this study, we examined techno-overload from the perspective of its potential links to employees' relationships with work, the organization, and the profession. We analyzed the relationships between techno-overload and employees' intention to leave the organization (turnover intention) and two types of insecurity related to current employment and external labor market – job and occupation insecurity.

Technological changes require employees to develop and acquire new knowledge and skills, plan and organize work effectively, and adapt psychologically to these changes (Fuchs and Najmaei, 2023). In the context of techno-overload, difficulties in adaptation or unsatisfactory work results may be associated with anxiety and fears that professional qualifications become insufficient to master the job, that the job will not be done properly and may be lost, and even that the occupation may change with technological progress and become less stable and secure in the labor market.

### 2.2 Theoretical background

The theoretical framework of this study integrates the Job Demands–Resources and the Social Exchange theories. Two theories are used as complementary explanations, as they address different aspects of workers' reactions to technological overload. The Job Demands–Resources theory explains the stress-inducing nature of technological overload as a job demand and its role in workers' perceived employment-related insecurity, while Social Exchange Theory accounts for how workers respond to these adverse conditions over time through cognitive evaluation and behavioral intentions to leave.

The Job Demands-Resources Theory is one of the most widely recognized, research-based, and empirically supported theories explaining the relationships among the digital work environment, employee health impairment, motivational processes, and employee or organizational outcomes (Scholze and Hecker, 2024). The motivational process originates from job resources (autonomy, feedback, social support, developmental opportunities)—elements of work that energize and motivate employees and support goal achievement and personal development. The health impairment process arises from high or poorly designed cognitive, emotional, or workload-related job demands, which take physical or psychological costs, including fatigue, disengagement, health problems, and poor work results (Bakker et al., 2023; Demerouti, 2024).

Every job position or work role is characterized by the job demands placed on it and the external (job-related) and internal (personal) resources required to meet these demands (Demerouti et al., 2001). The application of digital technologies may not affect the content of the job role or the structure of tasks, but it inevitably affects the volume and pace of work performed (Bakker and Demerouti, 2007, 2017). Following the logic of health impairment (Bakker et al., 2023), the increasing technological demands of jobs may become a source of technostress (Bunjak et al., 2021), resulting in feelings of unpredictability and a lack of confidence in one's ability to perform and control the work process. Unsuccessful efforts to meet high technology-related job demands may contribute to feelings of exhaustion, stress, and, further, to lower productivity, reduced job satisfaction (Ragu-Nathan et al., 2008; Suh and Lee, 2017), lower employee creativity (Ren et al., 2025), increased work–life conflict (Harris et al., 2022), or negative emotions (Lee, 2016). As a result, this can create feelings of insecurity about maintaining a job, increase doubts about career prospects in the current organization, and intensify anxiety about the impact of technology-induced changes on the profession in the external labor market.

The Social Exchange Theory (Blau, 1964; Cropanzano and Mitchell, 2005; Stana and Nicolajsen, 2024) examines work relationships in terms of mutual obligations, norms of fairness, and reciprocal exchange between employers and employees. Workers remain committed to the organization when the rewards (such as support, resources, and fair treatment) are balanced with the costs, including time and effort. These principles are applied in the Psychological Contract (Rousseau et al., 2018) and the Perceived Organizational Support theories (Rhoades and Eisenberger, 2002). For example, psychological contract, as it is defined by Rousseau et al. (2018), means “a cognitive schema, or system of beliefs, representing an individual's perceptions of his or her own and another's obligations, defined as the duties or responsibilities one feels bound to perform” (p. 1081). Regarding perceived organizational support, Eisenberger et al. (1986) proposed that “employees develop global beliefs concerning the extent to which the organization values their contributions and cares about their wellbeing” (p. 501).

Techno-overload creates conditions for increased demands, which, according to the principles of perceived organizational support, must be balanced with necessary support for work, opportunities to strengthen professional skills, measures to ensure employee wellbeing at work, and help in maintaining psychological and professional balance. This is a social exchange situation in which employers' expectations that employees will successfully adapt to digital requirements and the growing technological load must be met with obligations and measures that provide the right conditions for fulfilling these expectations and also address employee expectations related to technological innovations. As Rhoades and Eisenberger (2002) state: “To the extent that both the employee and the employer apply the reciprocity norm to their relationship, favorable treatment received by either party is reciprocated, leading to beneficial outcomes for both” (p. 698). When technological challenges are perceived as excessive demands and added responsibilities without adequate support, workers may prioritize their own interests and wellbeing over those of the organization. When this exchange is unbalanced, it may be interpreted as an organizational failure to uphold a social contract. Thus, workers respond by reducing their work contributions, psychologically detaching from tasks, putting in minimal effort, or even planning to leave the organization (Wayne et al., 1997; Topa et al., 2022).

Integrating both theoretical approaches enables us to link internal psychological strain processes to relational workplace outcomes. The Job Demands-Resources Theory describes how techno-overload functions as a job demand that depletes employees' inner resources, thereby contributing to perceptions of job and occupation insecurity. The Social Exchange Theory perspective provides theoretical principles that help explain how employees interpret excessive technological demands within the employee–organization exchange relationship and why one reaction may be the intention to leave the organization.

### 2.3 Research hypotheses

*Techno-overload and job insecurity*. “Stable work” usually refers to secure, predictable, and permanent employment, and from the employee's perspective, it typically involves the expectation of having a job for a specific period, ideally in the near future. However, nowadays it is a conditional term, as the world of work is dynamic and shaped by objective processes of organizational development, the variety of employment contracts, and improvements in work processes and methods. Not only are workplace requirements constantly changing, but so are the content of professions and their diversity, to which one must adapt, improve professional competencies, and plan a professional career, taking into account the dynamics of the labor market. In this way, various external, objective factors can affect employees' work-related insecurity.

Subjectively felt insecurity differs from the insecurity experienced when a person loses a job (Sverke and Hellgren, 2002). As De Witte et al. (2016) point out, subjective job insecurity is significant, as anxiety about future uncertainty and potential job loss has a stronger impact on a person's psychological wellbeing than the actual situation of dismissal. In this study, we follow the job insecurity model, which encompasses both quantitative and qualitative types of insecurity. Quantitative job insecurity refers to concern about losing the job itself, whereas qualitative insecurity involves worrying about the likelihood of losing valued aspects of the job (De Witte et al., 2010). We examined a quantitative type of job insecurity, along with turnover intention and occupation insecurity.

Technological progress can be a significant determinant of job insecurity, as employees may feel overwhelmed by high technological demands and excessive reliance on technology, which may relate to anxiety about retaining their jobs. Research in this area, as noted by Gallie et al. (2017), is still in its early stages, with only a few studies examining the links between technological aspects of work and job insecurity. Gallie et al. (2017) analyzed the relationship between digital technologies and subjective job insecurity, finding that employees in high-tech organizations experience greater job insecurity regarding the retention of their positions. The authors believe that automation and the resulting decrease in traditional work tasks can exacerbate subjective job insecurity. A study by Ghani et al. (2022) that included employees in Pakistan's retail industry found a strong relationship among work-related stress, technological advancements, and job insecurity.

Although previous research has focused on technological overload as a work-related stressor, its implications extend beyond its direct effects on strain. Excessive technological demands may undermine employees' confidence in their ability to meet changing job requirements, leading to doubts about their ability to retain their current jobs. Technological overload may therefore be a precursor to job insecurity. Understanding this relationship is important because perceptions of job insecurity may be consistently associated with employees' intentions to leave their organizations and anxiety of the occupation insecurity in the labor market. In this study, following the Job Demands-Resources theory, we expected that in the digital work environment, workers may feel high levels of job insecurity due to fear of becoming unable to adapt to technological demands, and we examined techno-overload as a potential factor in job insecurity.

H1. Techno-overload is positively related to employee job insecurity.

*Techno-overload and turnover intention*. According to Carmeli and Weisberg (2006), the term “turnover intention” refers to three elements in the withdrawal cognition process—thoughts of quitting, the intention to search for another job, and the intention to quit, but not to the turnover itself (p. 193). The intention to quit is defined as both an attitude and a form of withdrawal behavior (Marsh et al., 2022). This attitude primarily reflects a cognitive and behavioral response to the growing technological load and changing technology-related job demands. It may be related to lower involvement in work or even “quiet quitting,” in which employees do not terminate their employment relationship with the organization but have minimal commitment to work, distance themselves from it due to problems caused by high workloads, unsatisfactory working conditions, or a lack of opportunities to reconcile work with personal life (Bernuzzi et al., 2025). Those who intend to leave do not always do so; however, research indicates that this intention is directly related to actual departure from the organization and is a significant factor in employee turnover (Cohen et al., 2016).

The reasons why workers leave an organization can be diverse (Belete, 2018), ranging from low job satisfaction (Le et al., 2023) to an unsatisfactory social climate, such as bullying (Sun et al., 2025). The type of work arrangement, for example, telecommuting, may also be important. Telecommuting is a form of remote work that offers flexible work locations and enables employees to perform their tasks outside the traditional workplace. Gajendran and Harrison (2007) reported in a meta-analytic study that telecommuting was associated with lower turnover intention. On the other hand, remote work can be sensitive, as it may create a sense of professional isolation. Bermúdez-González et al. (2024) examined the relationships between professional isolation and organizational commitment among employees working in a teleworking modality in the technological sector in Spain. The results show that professional isolation negatively affects organizational commitment. This may relate to a higher intention among employees to leave the organization (Guzeller and Celiker, 2020).

However, as Carlson et al. (2017) note in a systematic review, research on the relationship between techno-overload and turnover intention remains limited. In this context, research linking intention to leave to job characteristics, specifically digital job demands, is relevant. Marsh et al. (2022)), in one of the most recent reviews of digital workplace research, conclude that intention to leave is one of the outcomes, along with low productivity, performance, and organizational commitment.

If technological changes are not significant, the organization provides appropriate support and implements appropriate work arrangements, and the employee successfully adapts without incurring major personal costs. Changes can then be accepted without negative emotional reactions or behavioral decisions. For example, telecommuting, a form of remote work that offers flexible work locations and allows employees to perform their tasks outside the traditional workplace, may be a direct or contextual factor in reducing employees' turnover intention.

However, when technology-related job demands significantly exceed those of previous working conditions, doubts may arise over time about the employer's fulfillment of its obligations. According to the Social Exchange Theory, perceived imbalance adjusts perceptions of how the employer adheres to the principles of fairness and reciprocal exchange. This may encourage consideration of the value of work in the organization, highlight a growing imbalance between the employer's requirements and personal contributions, and bring the intention to seek work outside the organization closer to realization. Employees who find technological demands excessive or destabilizing may consider leaving as a way to regain control, reduce stress, or protect their wellbeing (Califf and Brooks, 2020). Thus, we expected a positive relationship between techno- overload and turnover intention.

H2. Techno-overload is positively related to employee turnover intention.

*Techno-overload and occupation insecurity*. In this study, we included a new and previously understudied phenomenon, occupation insecurity, as one of the consequences of techno-overload. Occupation insecurity arising from technological development complements the spectrum of phenomena describing employee insecurity in the labor market and can provide more empirical evidence confirming the influence of digital technologies, specifically techno-overload, on employment insecurity. In 2023, the first publication introduced the concept of occupation insecurity, defining it as “...people's fears about the future of their occupations due to technological advancements” (Roll et al., 2023, p. 2).

Uncertainty about the profession's future is reinforced by rapid technological progress, which, according to Mokyr et al. (2015), raises concerns that new digital technologies may replace certain jobs and lead to technological unemployment. Rapid changes create uncertainty and anxiety about how professions can evolve, which competencies need to be strengthened to find jobs in other professions also affected by technology, and whether current jobs will be lost. When planning a professional career, it is no longer enough to focus solely on career plans within a specific organization; it is necessary to consider occupational changes and develop competencies that meet the increasing technological requirements of employers in the labor market (Susskind and Susskind, 2018).

Roll et al. (2023) introduced the occupation insecurity model and developed the Occupation Insecurity Scale (OCIS), which combines global and content-specific occupation insecurity sub-dimensions (Roll et al., 2023). Global occupation insecurity refers to people's fear about the future of their occupation: that a concrete profession may no longer be needed, may disappear, and that they may need to switch to another occupation. Content occupation insecurity refers to employees' anxiety that technological innovations will significantly alter how they perform their jobs, the responsibilities they hold, and the competencies they require. The authors empirically confirm the validity of the method and provide data showing that occupation insecurity has positive relationships with employee burnout and negative relationships with work engagement (Roll et al., 2023). Later, this instrument was cross–nationally validated in Germany, the USA, Belgium, and China (Roll et al., 2025). In all samples from these countries, Global occupation insecurity was positively associated with burnout and physical health complaints, and negatively correlated with self-rated performance.

In developing research on occupation insecurity, it is relevant to examine not only its consequences but also its predictors to reveal the significance of the work environment, including technological characteristics, for the perception of occupational insecurity. So far, this is a very little-studied area; we have not found a single study that examines the links between work characteristics related to the technological environment and occupation insecurity. Theoretically, we based the link between techno-overload and occupation insecurity on the Job Demands-Resources theory, which posits that techno-overload, as a high job demand, may become a source of health impairment, stress, or burnout, and of distal outcomes such as occupation insecurity. In this study, we expected that techno-overload, a job demand arising from digitalization, could contribute to employee insecurity not only about the possibility of keeping their jobs (job insecurity) but also about future changes in their occupations (occupation insecurity).

H3. Techno-overload is positively related to employees' occupation insecurity.

*The mediating role of job insecurity*. The links between techno-overload and various consequences may not be direct; this techno-stress creator may relate to distal consequences (intention to leave and occupation insecurity) through intermediate employee states. Research reveals that job insecurity is a reaction to digital technologies, as a source of techno-stress (Gallie et al., 2017; Ghani et al., 2022). Meanwhile, intentions to leave the organization and fears of occupation insecurity form over a longer period of time. Research confirms the detrimental consequences of job insecurity on employee turnover intentions (Lee et al., 2018; Staufenbiel and König, 2010). Positive relationships were found between quantitative job insecurity and both types of occupation insecurity - global and content (Roll et al., 2023). Numerous studies have examined job insecurity as a direct predictor of negative employee outcomes, whereas only a few have examined its role as an intermediate variable. For example, in a study of nurses, Bitmiş and Ergeneli (2015) found that job insecurity mediated the relationship between psychological capital and burnout. Park and Ono (2017) found that exposure to workplace bullying negatively predicted work engagement and health among Korean employees, with this link mediated by high perceived job insecurity. Sharif et al. (2025) conducted a three-wave online survey of employees at chain restaurants in the People's Republic of China, confirming that job insecurity mediated the relationship between the use of artificial intelligence and employee wellbeing and career success. Therefore, in this study, we investigated the role of techno-overload in the above-discussed consequences in two ways: we examined the associations between techno-overload and job insecurity, turnover intention, and occupation insecurity, and, moreover, we sought to reveal indirect relationships between techno-overload and intention to leave and occupation insecurity. Job insecurity, as a proximal reaction to technological overload, is examined as a potential mediator explaining these links. Employees' responses to techno-overload-induced job insecurity may include an intention to leave the organization and, more broadly, a perceived occupation insecurity in the future. A higher level of techno-overload is likely to be positively related to job insecurity—a perceived risk of job loss, which is expected to be linked to a stronger desire to leave the organization and to fears of future occupation security. The Social Exchange Theory suggests that employees evaluate the quality of their relationships with the organization based on perceived support, fairness, and reciprocity. When technology-related demands raise concerns about job continuity and future career prospects, employees may believe the organization is not adequately protecting their interests. These perceptions may be linked to lower organizational commitment and a higher intention to leave. In this process, job insecurity may mediate the relationship between techno-overload and employee turnover intentions.

H4. Employee job insecurity mediates the relationship between techno-overload and turnover intention.

Furthermore, the JD-R theory provides the basis for the hypothesis of positive indirect relationships between techno-overload and occupation insecurity. Managing techno-overload as a technology-related job demand requires employees to allocate additional cognitive, emotional, and time resources. When these demands exceed available resources, tension and uncertainty may arise, increasing job insecurity, which may be positively related to the downstream outcome—occupation insecurity and may act as a mediator, through which techno-overload may be indirectly related to occupation insecurity.

H5. Employee job insecurity mediates the relationship between techno-overload and perceived occupation insecurity.

In general, job insecurity serves as a bridge between processes that describe indirect relationships between technology-induced overload and two types of employment-related outcomes—intention to leave the organization and potential occupation insecurity in the labor market.

### 2.4 The current study

**Figure 1:**
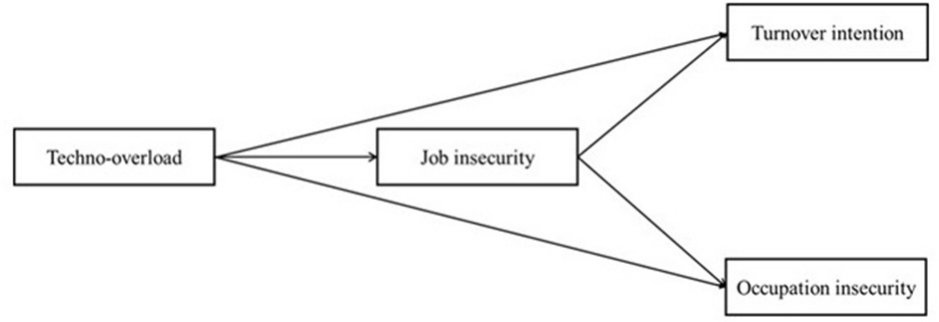
The research model.

To summarize, this study aims to investigate the role of techno-overload in relation to employee job insecurity, turnover intention, and occupation insecurity among Lithuanian employees. The conceptual framework for understanding the employment-related consequences of techno-overload integrates the Job Demands-Resources and Social Exchange theories. The Job Demands–Resources theory emphasizes the resource-based strain pathway and explains how digital job demands may be associated with greater job and occupation insecurity. The Social Exchange Theory describes turnover intention as an employee response to overload, perceived as a breach of the employer's obligations to provide a healthy and supportive work environment and to offer necessary support when needed. The research model is presented in Figure 1. It suggests that techno-overload is related to job insecurity, turnover intention, and occupation insecurity, and that job insecurity mediates the relationship between techno-overload and both turnover intention and occupation insecurity.

## 3 Materials and methods

### 3.1 Research procedure and sample

Empirical data were collected in 2025 using a cross-sectional research design. Data were collected in Lithuania in collaboration with a market and public opinion research company. The survey was conducted using Computer-Assisted Telephone Interview (CATI) and Computer-Assisted Web Interviewing (CAWI). A sample of this study comprised 754 employees from various Lithuanian organizations who reported using information technologies at work. Our sample consisted of 341 (45.2%) men and 413 (54.8%) women, with ages ranging from 19 to 64 years (*M* = 41.9, *SD* = 12.06). 533 (70.7%) individuals have higher university education, while 221 (29.3%) have vocational or secondary education. 306 (40.6%) worked in the public sector, 431 (57.2%) in the private sector, and 17 (2.3%) in the non-governmental sector. Regarding the position, 212 (28.1%) of the study participants held managerial positions, while 542 (71.9%) held non-managerial positions. Length of organizational tenure ranged from 1 to 44 years (*M* = 9.4; *SD* = 9.09).

The research was conducted in accordance with the ICC/Esomar International Code on Market, Opinion and Social Research and Data Analytics (ICC/ESOMAR International Code on Market, Opinion and Social Research and Data Analytics, 2025). Participants were informed that the study was conducted in accordance with local legislation, institutional requirements, and research ethics requirements, that their participation was voluntary, and that they could withdraw from the study at any time. Informed consent for participation in the study was obtained from each participant before data collection. The anonymity and confidentiality of the study participants were ensured. Participants were informed that their personal information would not be disclosed when responding and that the results would be analyzed in aggregate and for scientific purposes only.

### 3.2 Research instruments

*Techno-overload* was assessed using a five-item scale (“I am forced by this technology to do more work than I can handle”) developed by Ragu-Nathan et al. (2008). Techno-overload refers to situations where information and communication technologies compel employees to work faster and for longer periods. The exploratory factor analysis with Varimax rotation confirmed a one-factor scale structure (KMO = 0.755, Bartlett's test of sphericity: Chi-square = 938.2, df = 6, *p* < 0.001), explaining 62.5% of the variance, with factor loadings ranging from 0.769 to 0.833.

*Job insecurity* was measured using three items from the Quantitative Job Insecurity Scale (“I think I might lose my job in the near future”) initially developed by De Witte (2000, as cited in De Witte et al., 2010) and validated in many countries (Vander Elst et al., 2014; Urbanaviciute et al., 2021). The scale assesses employees' anxiety about the possibility of losing their jobs at their current organization in the near future. The exploratory factor analysis with Varimax rotation confirmed a one-factor scale structure (KMO = 0.789; Bartlett's test of sphericity, Chi-square = 2077.6, df = 6, p < 0.001), explaining 76.8% of the variance, with factor loadings ranging from 0.820 to 0.919.

Turnover intention, which reflects employees' likelihood of leaving the organization, was evaluated using the four-item Intentions to Quit scale (“As soon as I can find a better job, I'll leave this company”) created by Wayne et al. (1997). The exploratory factor analysis with Varimax rotation confirmed a one-factor structure (KMO = 0.835, Bartlett's test of sphericity Chi-square = 1953.7, df = 6, *p* < 0.001), explaining 77.5% of the variance, with factor loadings ranging from 0.828 to 0.916.

Occupation insecurity which reflects the employee's fear that the occupation may undergo significant changes due to technological advancements (such as automation, innovative technologies, artificial intelligence, and robotics) was assessed using the four items from the Global Occupation Insecurity scale developed by Roll et al. (2023) (“I am worried that my occupation will not be needed anymore in the future due to the advancement of technology”). We checked for the scale's construct validity by the exploratory factor analysis, which confirmed the one-factor structure of the scale (Varimax rotation, KMO = 0.755, Bartlett's test of sphericity: Chi-square = 938.2, df = 6, *p* < 0.001), accounting for 62.5% of the variance with factor loadings ranging from 0.592 to 0.695.

### 3.3 Statistical analyses

For this study, data analysis was conducted using IBM SPSS version 21 and IBM SPSS AMOS version 21. First, we examined the scales' construct validity and reliability, followed by descriptive statistics of the research variables (means, standard deviations, Pearson correlations, and Student's *t*-test). To assess the scales' reliability, Cronbach's alpha coefficients were calculated, and construct validity was evaluated using exploratory factor analysis. Structural equation modeling (SEM) with 5,000 bootstrap resamples was conducted to test the hypothesized structural relationships, including the direct effects of techno-overload on job insecurity, turnover intention, and occupation insecurity, and the mediating effect of job insecurity on the relationships between techno-overload and employee outcomes—turnover intention and occupation insecurity. Age, gender, and education were included as covariates. Model fit was assessed using multiple fit indices, including the chi-square statistic (χ^2^), the comparative fit index (CFI), the Tucker–Lewis index (TLI), and the root mean square error of approximation (RMSEA), with 90% confidence intervals. Bootstrapping with 5,000 resamples was used to generate bias-corrected 95% confidence intervals for indirect effects. The indirect effect through the mediating variable was confirmed if its 95% confidence interval did not include 0.

Harman's single-factor test was conducted to assess potential common-method bias in the dataset. The unrotated exploratory factor analysis using principal axis factoring was performed for all items, which were grouped into a single factor. It explained 36% of the total variance. Since this is below the 50% threshold, the common method bias is not considered a significant concern in the data (Podsakoff et al., 2024). We also conducted a single-factor CFA model in AMOS with all items loading on a single latent factor, which showed poor fit (χ^2^/df = 35.33; CFI = 0.440; TLI = 0.360; RMSEA = 0.214). A four-factor measurement model provided an acceptable model fit (χ^2^/df = 2.65; CFI = 0.978; TLI = 0.971; RMSEA = 0.047). Cronbach's alpha coefficients for all variables exceeded 0.88, reflecting good internal reliability of the scales. They are shown in Table 1.

**Table 1:** Means, standard deviations, and Pearson correlations between study variables (*N* = 754).

Variables	*M*	*SD*	1	2	3	4	5
Techno-overload	2.90	0.87	(0.894)				
Job insecurity	2.49	0.98	0.334^**^	(0.896)			
Turnover intention	2.93	1.02	0.193^**^	0.477^**^	(0.903)		
Occupation insecurity	3.23	0.77	0.491^**^	0.293^**^	0.124^**^	(0.797)	

Cronbach's alpha coefficients are presented on the diagonal. ^**^p < 0.01. *M*, mean, *SD*, standard deviation.

## 4 Results

### 4.1 Descriptive statistics

Table 1 presents the means, standard deviations, and Pearson correlation coefficients between the study variables.

Significant positive correlations were found between all study variables. The dependent variable, turnover intention, was most strongly correlated with job insecurity (*r* = 0.477, *p* < 0.01), while occupation insecurity was most strongly associated with techno-overload (*r* = 0.491, *p* < 0.01). When comparing the values of the studied variables in groups by gender and education level, no significant differences were found. Age was positively associated with job insecurity (*r* = 0.091, *p* < 0.05) and negatively associated with turnover intention (*r* = −0.124, *p* < 0.01); however, these associations were weak. Comparing the means of variables obtained in the groups of public and private sector employees, it was found that those working in the public sector rated techno-overload in their work higher than those working in the private sector (respectively *M* = 3,02, *SD* = 0.87, and *M* = 2.81, *SD* = 0.89, *t* = 3.19, df = 735, *p* < 0.001). The means of the other variables in these groups were not statistically significant. Techno-overload was the only variable that differed when comparing groups by position: respondents in managerial positions rated techno-overload in their work higher than employees in non-managerial positions (*M* = 3.02, *SD* = 0.83, and *M* = 2.86, *SD* = 0.90, *t* = 2.22, df = 752, *p* = 0.013, respectively). Tenure in the organization was positively but weakly associated with occupation insecurity (*r* = 0.08, *p* < 0.05) and negatively correlated with intention to leave the organization (*r* = −0.117, *p* < 0.01).

Summarizing the relationships between demographic characteristics and the variables analyzed, the observed correlations are generally weak. No significant differences were observed in the means across gender and education groups. As age increases, job insecurity—employees' anxiety about losing their jobs—increases, but their intention to quit from the current job decreases. Employees with longer tenure in the organization are also less likely to quit their current job. Those in the public sector and in managerial positions rate techno-overload higher than workers in the private sector and in non-management positions.

### 4.2 Hypothesis testing

Hypotheses were tested using Structural Equation Modeling. A path model included linear relationships among techno-overload and job insecurity, turnover intention, and occupation insecurity, as well as mediating relationships between techno-overload and the two dependent variables—turnover intention and occupation insecurity, with job insecurity as a mediator. The model, which accounted for all the above-mentioned linear and mediating relationships, was confirmed: χ^2^ (1) = 1.19, CFI = 1.00, TLI = 0.998, RMSEA = 0.016, CI [0.00; 0.100]. However, the direct link between techno-overload and turnover intention was not significant (β = 0.038, *p* = 0.265); therefore, we removed this link from the model. The modification resulted in a final model with a good overall fit: χ^2^(2) = 2.34, *p* = 0.296, CFI = 0.999, TLI = 0.997, RMSEA = 0.017, CI [0.00; 0.076]. Although both models were significant, given the parsimony principle, the second model, which excluded the insignificant linear relationship between techno-overload and turnover intention, was selected for further analysis.

Theoretically, this modification can be justified by the conceptual research assumptions discussed earlier. Consistent with the Social Exchange Theory, employees evaluate their relationship with the organization based on the balance between changing demands and organizational support, or between employee contributions and inducements. However, the insignificant direct link between techno-overload and turnover intention suggests that this relationship may be more complex, with various work-environment and individual factors acting as mediators or moderators (Climek et al., 2024). Our study revealed that techno-overload appears to be linked to employees' turnover intentions via job insecurity. When employees perceive that, under conditions of technological load, the organization is unable or unwilling to provide adequate employment security in exchange for their efforts, they respond with withdrawal cognitions that manifest as turnover intentions. Therefore, the insignificant direct link indicates that the relationship between techno-overload and turnover intention operates primarily through job insecurity rather than a direct exchange.

**Figure 2:**
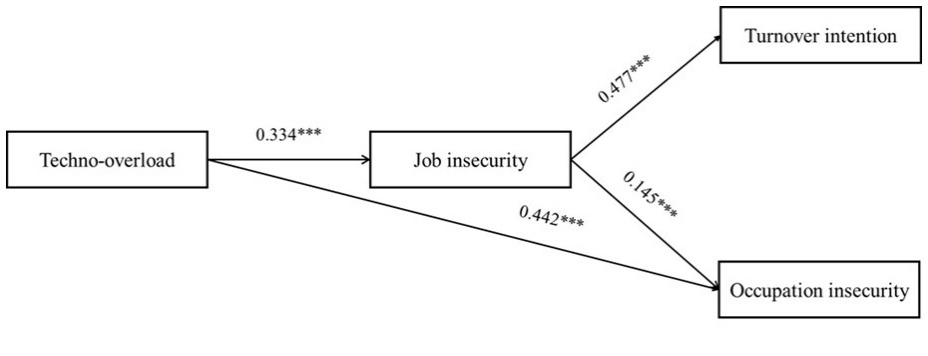
Direct and indirect standardized effects of techno-overload on job insecurity, turnover intention, and occupation insecurity. ***p > 0.001.

Figure 2 presents the final empirical model, illustrating the empirically supported direct and indirect relationships among the variables. Table 2 presents standardized regression weight estimates, along with confidence intervals, describing the total, direct, and indirect effects of techno-overload and job insecurity on turnover intention and occupation insecurity.

**Table 2:** Standardized effects of techno-overload and job insecurity in predicting turnover intention and occupation insecurity.

Independent variable	Mediator	Dependent variable	Direct effect	Indirect effect	Total effect
			β, 95% CI	β, 95% CI	β, 95% CI
Techno-overload	Job insecurity	Turnover intention	0.000	0.159^***^ [0.120, 0.204]	0.159^***^ [0.120, 0.204]
		Occupation insecurity	0.442^***^ [0.365, 0.512]	0.048^***^ [0.024, 0.078]	0.491^***^ [0.424, 0.553]

^***^p < 0.001. *β*, standardized beta coefficients; 95% CI, confidence intervals.

The results showed that techno-overload is directly associated with job insecurity (β = 0.334, *p* < 0.001) and occupation insecurity (β = 0.442, *p* < 0.001), supporting H1 and H3. Techno-overload correlated with turnover intention (*r* = 0.193, *p* < 0.01; see Table 1); however, this relationship was weak, and in the final model, only the indirect link of techno-overload on turnover intention via job insecurity was significant. Therefore, H2, which posits that techno-overload is positively related to employee turnover intention, was not confirmed. Job insecurity positively predicted turnover intention (β = 0.477, *p* < 0.001), as well as occupation insecurity (β = 0.145, *p* < 0.001).

Although the direct link between techno-overload and turnover intention was not confirmed, a significant indirect relationship was established through job insecurity as a mediating variable (β = 0.159, *p* < 0.001, CI [0.120, 0.204]). Techno-overload, together with job insecurity, accounted for 23.0% of the variance in turnover intention. Thus, the fourth research hypothesis (H4), stating that employee job insecurity mediates the relationship between techno-overload and turnover intention, was confirmed.

Another aspect of techno-overload emerged when analyzing the link between this type of job demand and a phenomenon directly unrelated to work - occupation insecurity. As mentioned above, a sufficiently strong link between techno-overload and occupation insecurity has been established. The data in Table 2 show that the direct effect of techno-overload on occupation insecurity was supplemented by an indirect effect through job insecurity as a mediating variable (β = 0.048, *p* < 0.001, CI [0.024, 0.078]). Job insecurity mediated the relationship between employees' evaluations of techno-overload at work and occupation insecurity, supporting H5. Techno-overload, together with job insecurity, accounted for 26.0% of the variance in occupation insecurity. In this way, higher techno-overload was associated with greater assessment of occupation insecurity directly and via greater job insecurity, which mediates this relationship.

## 5 Discussion

The development of digital technologies is an essential feature of the “new normal” in the world of work. However, when the requirements associated with these technologies exceed an employee's capabilities and the support provided by the organization or personal resources is insufficient, this van become a source of technostress, an increasingly studied phenomenon (Ragu-Nathan et al., 2008; Pansini et al., 2023).

The present study focuses on one of the technostress creators - techno-overload—and its role in relation to employee job insecurity, turnover intention, and occupation insecurity among a sample of Lithuanian employees. Techno-overload, as indicated by Tarafdar et al. (2007), refers to the increasing pace and workload resulting from the application of technology, which is one of the most significant contributors to employee stress (La Torre et al., 2019). Quantitative job insecurity refers to concern about the loss of the job (De Witte et al., 2010), turnover intention reflects the person's attitude and readiness to leave the organization (Marsh et al., 2022), and occupation insecurity refers to people's fears about the future of their occupations due to technological advancements (Roll et al., 2023, p. 2).

The results of our study align with two theoretical perspectives: The Job Demands-Resources Theory (Bakker and Demerouti, 2017) and the Social Exchange theory (Blau, 1964). According to the principles of the Job Demands-Resources Theory, techno-overload refers to a technological load that, similar to high general workload, exceeds optimal limits and employees' capabilities to meet these requirements (Carlson et al., 2017). Employees may perceive their job positions as insecure and may become concerned about keeping their jobs. Meanwhile, the principles of Social Exchange Theory explain the underlying processes that shape reactions to technology-related requirements. When the workload exceeds the job and personal resources, it may be perceived as a violation of the social contract and the principle of the equal and fair reciprocal exchange between employer and employee (Wayne et al., 1997). It may result in employees' intentions to leave the organization, or even in worries that the profession may change in the future due to technological innovations. From the perspective of Social Exchange Theory, employers' unbalanced demands and obligations, without adequate leadership, working conditions, or development opportunities, may create a work environment that supports tension and conflict in the labor relationship. In this inevitable work context, employees may perceive working conditions as unfair (Rousseau et al., 2018) and may believe that work no longer holds much value and is not worth making a significant effort.

We analyzed two types of links between techno-overload and job insecurity, turnover intention, and occupation insecurity. The first one reveals the associations between techno-overload and the aforementioned consequences. We hypothesized that techno-overload positively relates to job insecurity (H1), employee intention to leave the organization (H2), and occupation insecurity (H3). The second aspect emphasizes the indirect relationships between techno-overload, turnover intention, and occupation insecurity. Job insecurity can be a short-term reaction to techno-overload, while turnover intention and occupation insecurity develop over a longer period and may be related to both techno-overload and job insecurity. Therefore, job insecurity is analyzed as a mediating variable, highlighting the indirect relationships between techno-overload and turnover intention (H4) and between techno-overload and occupation insecurity (H5) via job insecurity.

The empirical data supported two of the three stated hypotheses regarding the direct relationships between techno-overload and the outcomes analyzed. It was found that techno-overload positively related to job insecurity (β = 0.334, *p* < 0.001) and occupation insecurity (β = 0.442, *p* < 0.001), supporting H1 and H3 (see Table 2 and Figure 2). Higher techno-overload was associated with greater job and occupation insecurity, and its association with turnover intention appears to be indirect, operating through job insecurity. The positive relationship between techno-overload and job and occupation insecurity can be explained by the principles of the Job Demands-Resources Theory: an employee can cope with high job demands if they are imposed for a short time. However, over time, as physical, mental, and other internal resources are depleted, work may become a constant source of strain and burnout (Demerouti et al., 2001). Under these conditions, if the organization's support remains insufficient, feelings of vulnerability, uncertainty, and further insecurity may arise, and this imbalance may be interpreted as a threat to further work or career stability (Shoss, 2017).

However, the H2 was not supported, as techno-overload was not directly associated with employee intention to quit. The correlation between these variables was positive but weak (*r* = 0.193, p < 0.01; see Table 1), and this relationship was no longer significant in the final model (β = 0.038, *p* = 0.265). One explanation may be that a relatively small number of respondents (22.7 percent) answered positively to the question “Are you currently looking for a job in another organization?” The mean of the turnover intention variable was also not high (*M* = 2.93, *SD* = 1.02, see Table 1). This indicates that the majority of participants in our study were not actively involved in job search activities.

It is also important to note that the intention to leave the organization may be driven not only by workplace changes related to technological overload but also by other organizational, personal and contextual factors: an unsatisfactory team climate, job dissatisfaction, limited opportunities for advancement, perceived weak employability following a change of residence, or individual differences in job search behavior (Baranchenko et al., 2020; Lazzari et al., 2022; Li and Yao, 2022). Authors of systematic reviews also emphasize that the relationship between technological workload and turnover intention may depend on various mediating phenomena or on interactions between techno-overload and contextual factors, including alternative employment opportunities, labor demand, or the economic situation in a particular region (Climek et al., 2024; Marsh et al., 2022).

Although the cross-sectional design enabled the examination of associations between techno-overload, job insecurity, occupational insecurity, and turnover intention, it does not permit conclusions about causal or temporal relationships. Perceptions of insecurity or turnover intentions may become factors in perceptions of technology-related load, and longitudinal studies could help reveal relationships in the opposite direction, thereby providing a basis for understanding the direction and dynamics of these relationships over time (Schaie, 1965).

Examining the indirect links between techno-overload (independent variable) and intention to leave and occupational insecurity (dependent variables), two hypotheses were formulated regarding the mediating role of job insecurity. We hypothesized that job insecurity mediates the relationships between techno-overload and turnover intention (H4) and between techno-overload and perceived occupation insecurity (H5). The results supported both of them (see Table 2 and Figure 2). Regarding turnover intention, although the direct relationship between techno-overload and intention to leave was non-significant, an indirect link emerged when job insecurity was included as a mediating variable. Job insecurity fully mediated the relationship between techno-overload and intention to leave. Regarding occupation insecurity, partial mediation was observed: after including job insecurity, the relationship between techno-overload and occupation insecurity weakened but remained significant (see Table 2 and Figure 2). This result is consistent with the Job Demands-Resources Theory, which states that a person responds to external factors in the work environment (in our study, techno-overload) primarily by experiencing internal states (here, job insecurity), which relate to further consequences.

Employees who rate techno-overload higher and feel less secure in their jobs have a stronger intention to leave the organization and may feel higher anxiety that their profession may change or even disappear due to technological progress. Those working in environments with increased technological demands, where work requires a faster pace and larger tasks, may experience greater insecurity due to the risk of losing their jobs if they do not adapt and cope effectively. Moreover, our study reveals that the undesirable consequences of techno-overload and job insecurity are not limited to the work role and can spill over (transfer) into perceptions of future technology-related changes in their professions. The digitalization and other technological innovations may make working in the same profession more challenging in the future. The Job Demands-Resources Theory emphasizes that personal resources, such as self-efficacy or psychological capital, are crucial for the relationship between job demands and various outcomes (Xanthopoulou et al., 2007; Bakker et al., 2023). Job security can also be a personal resource, but in the environment of techno-overload or pressure of other technological factors, it can shift to the opposite state – job insecurity. This not only disrupts work but may also be associated with further undesirable consequences – including an intention to leave the organization and feelings of anxiety related to reduced occupational security. The results of our study align with research that identifies perceived job insecurity as an intermediary variable in relationships among various work-related phenomena (Bitmiş and Ergeneli, 2015; Park and Ono, 2017; Sharif et al., 2025).

## 6 Limitations and guidelines for future studies

The study's limitations relate to several aspects. First, the quota sample of Lithuanian employees was selected based on age, gender, education, and place of residence. When comparing the groups across these demographic characteristics, no significant differences were found in any of the research variables. This sample is not representative of the sector in which the organization operates, the employee's position, or the profession. Therefore, future studies on techno-overload, job insecurity, and turnover intention are relevant, with samples designed to capture a broader range of professional characteristics. This would demonstrate the extent to which the study's conclusions are applicable to employees across various professions, positions, and sectors.

Another limitation of the study is the reliance on a single source of data (information) – employee self-reporting, which, according to Brutus et al. (2013), poses threats to data validity. The researchers acknowledge that the interpretation of the obtained findings may be limited when results rely solely on participants' self-observation. Interview responses reflecting opinions, attitudes, or personal experiences can be influenced by personal factors, experiences, or situational features. To minimize subjective bias, we assessed the data for potential common-method bias (Podsakoff et al., 2024). It is also important to note that our study was cross-sectional, with data collected at a single point in time. However, perceptions of the constructs under analysis can change over time; they are not stable or fixed. A longitudinal research strategy, when studying the dynamics of employment-related insecurity or supplementing intentions to leave with actual employee turnover indicators over time, provides researchers with more opportunities to reveal the stability or dynamics of these phenomena. Additionally, for job and occupation insecurity, qualitative studies that include employees with diverse experiences and professions are relevant, creating a broader, reality-based picture of the content, assumptions, and consequences of these phenomena.

The analysis of scientific literature conducted in the context of our study highlighted that techno-overload can be a positive factor for work motivation and engagement (Li and Wang, 2021; Nascimento et al., 2024; Ren et al., 2025), but only when employees view techno-overload as a challenging technology-related demand and not as a hindrance. The phenomenon of techno-overload as a challenging job demand, and its links to current employment and future professional prospects in the external labor market, reveal an interesting and promising research direction.

Future research should focus on other techno-overload creators and a wider spectrum of stress-inducing factors in the digital environment to examine their interactions with job insecurity, turnover intention, and occupational insecurity, accounting for their intensity and combinations. Such studies are not new, but they remain underdeveloped. For example, Pflügner et al. (2024) emphasize that techno-stressors can reinforce one another, especially when intense; for instance, techno-overload and techno-uncertainty, acting together, are associated with greater job burnout.

There is no doubt that it is particularly relevant to develop research on a comparatively new phenomenon, occupation insecurity, and its links with the work environment and individual employee characteristics. This type of insecurity complements the range of insecurity constructs in the field of employment, alongside quantitative and qualitative job insecurity (De Witte et al., 2010; Urbanaviciute et al., 2021) and career insecurity (Roll et al., 2023, 2025). These studies are also relevant to newcomers starting their professional career or to students pursuing a profession. Insecurity about the future of every profession affects individuals' professional choices and career decision-making and, more broadly, may influence the balance of demand and supply of specialists across fields and other labor market processes. It can be directly related to career planning, evaluating future career prospects, and the wellbeing of individuals across family, personal, and work life, as well as to each individual's prospects in the labor market.

Stable personnel are the foundation for an organization's stable operation, and employees' intention to leave reflects their shifting attitudes toward work and the organization. Intention to leave is a way to cope with the fear of losing one's job, because it is easier for a working person to find another job than for someone who is unemployed. Investigating the factors driving employee turnover and the reasons for leaving remain relevant areas of study, despite decades of research. Digital working conditions encompass previously less significant or newly emerging technological factors that may be associated with employees' intention to leave. Our study reveals that one source of technostress, techno-overload, is positively associated with this intention, although the link was indirect and mediated by job insecurity. Various contextual and organizational factors, such as organizational support or learning opportunities, may also be important. Additionally, it is worth investigating the relationships of the techno-overload and job insecurity for other forms of employee withdrawal (Carlson et al., 2017), for example, for the phenomenon of “quiet quitting”, when employees do not terminate their employment relationship with the organization but have minimal commitment to work, distance themselves from it and put less efforts in it (Bernuzzi et al., 2025).

It is also important to note that our study was conducted with a sample of employees in Lithuanian public and private organizations. Despite rapidly evolving technological trends across countries, the generalizability of the results to organizations or labor markets in other countries is limited. Employees' perceptions of techno-overload and job and occupation insecurity may be shaped by broader institutional and economic conditions, including labor market and education policies, employment and social security systems, labor market flexibility, the intensity of digitalization, or opportunities for professional mobility (Goos, 2018). The relationships revealed in the study may differ across countries due to country-specific characteristics in economic security, technological development, and organizational support for digital adaptation. Although the results help explain employees' experiences in an increasingly digitized work environment, comparative international studies are needed to generalize them to other national contexts and determine whether the observed relationships remain stable across labor markets and organizational environments.

## 7 Implications

The majority of research on techno-stressors examines a composite indicator that combines techno-overload, techno-invasion, techno-complexity, techno-insecurity, and techno-uncertainty (Srivastava et al., 2015). Our study focuses on techno-overload and complements research on its associations with employee outcomes. La Torre et al. (2019), in their systematic review of technostress research, conclude that most studies combine various techno-stressors into a single indicator, obscuring more diverse and complex interrelationships. The authors emphasize that “studies should focus on a single aspect of technostress, rather than simultaneously approaching multiple stressors and consequences” (p. 32). This is especially important because assessing specific technostress creators provides an opportunity to identify their risks and explore ways to mitigate them.

The results contribute to this area by demonstrating the negative effects of techno-overload on employee outcomes and highlighting the value of studies that focus on the consequences of individual techno-stressors. More concretely, we aimed to contribute to the research on techno-overload by providing insights into its role in the insecurity of Lithuanian workers in the labor market and their intention to continue working in their current organization. To our knowledge, this is one of the first studies to combine job insecurity, turnover intention, and occupation insecurity as consequences of technology-related work overload and to reveal their interrelationships. The results complement research in this area in several ways: they extend studies on the relationship between techno-overload, as a technostress creator, and employee insecurity in the labor market; they identify the mediating role of job insecurity in linking techno-overload to turnover intention and occupational insecurity; and reveal the value of combining the Job Demands-Resources Theory and the Social Exchange Theory perspectives to examine and explain employee reactions to techno-overload in the digitalized work environment.

(1) The results extend previous research on techno-overload outcomes, revealing how techno-overload, as a technologically conditioned job demand, relates to employees' perceptions of job insecurity, their relations with the organization, and is positively associated with anxiety about future technology-related changes in the occupation.

(2) By examining the mediating role of job insecurity, the study explains the relationship between techno-overload and both turnover intention and occupational insecurity. The intention to leave a job is not just a subjective opinion; it is a crucial step in the process of making the final decision to leave and, subsequently, actually leaving the organization. The insecurity about the future of the occupation due to technological progress is also a significant concern for perceived individual employment prospects, as it partly reflects confidence in personal professional potential in the labor market and may be important for proactive career planning and implementation (Roll et al., 2023, 2025).

(3) The results also show that principles of the Job Demands-Resources Theory as well as the Social Exchange Theory may be helpful in explaining techno-overload relationships with analyzed employee outcomes and justifying the planning and implementation of new technologies at work. The findings expand the theory by showing that, in digital work environments, the analysis of workload, cognitive, or emotional demands, as presented in the Job Demands–Resources Theory (Bakker and Demerouti, 2017; Bakker et al., 2023), should include technology-related work characteristics, such as techno-overload, because technological factors have become a core part of many work roles. Digital job demands can affect employees' stress levels and job or occupation insecurity and, through social exchange processes, also shape their attitudes toward long-term organizational commitment, such as their intentions to stay or leave.

In practice, our study's findings suggest that Lithuanian organizations should ensure employees feel secure in their jobs and address factors that may undermine job security and positive long-term relationships with the organization. This is especially important as work processes improve with the increasing use of information technologies, which not only change how work is performed but also impose higher demands on employees' professional knowledge and skills, requiring them to be flexible and proactive in adapting to these changes. The dynamics and often complex nature of these innovations create digitalization-related challenges that can both motivate employees to improve technology-related competencies and contribute to anxiety about workplace stability and future employment.

Several aspects can be distinguished in this area. The first concerns the assignment of responsibility, in other words, who should be responsible for implementing digital technologies and adapting without negative consequences – the employee or the employer? There is no doubt that applying technologies depends more on optimizing work processes than on employees' wishes or even their current level of competence. However, to effectively manage these improvements, organizations should address a broader range of factors.

First, organizations should regulate workloads, conduct regular audits to assess whether employees are experiencing excessive technology-related demands, and identify sources of technological overload. Second, organizations should consider redesigning workflows and job responsibilities to ensure that technological demands remain manageable and do not exceed employees' available resources. Third, it is important not only to inform workers about these innovations but also to involve them in their implementation. Ongoing communication and discussion of changes to work processes and redesign may motivate employees to suggest creative solutions, thereby enabling them to adapt more easily to technological innovations. Fourth, it is essential to align the implementation of digital technologies with the opportunity for employees to work at least part of the time remotely—from home, co-working spaces, or other locations outside a traditional office setting, and to collaborate with colleagues and perform tasks using technology. Of course, this primarily depends on the job content, but where possible, remote work gives employees more opportunities to independently allocate tasks and working time and to regulate breaks for rest. Fifth, employees may perceive the technology-related load as too large, not because it is objectively so, but because digital tools are complex and require time to master fully, leaving workers without enough time to complete tasks within the usual time frame or without making mistakes. To avoid these negative consequences, two options arise: either increase staff numbers to reduce the technological load per employee, or strengthen employee competencies and create favorable working conditions without increasing personnel. However, it is not a choice between reducing techno-load and improving employee competencies, but rather about implementing interventions that enhance both. This helps employees master new work requirements and tools, adapt more easily to work processes, acquire the necessary knowledge, and develop professional skills. That's why employers should invest in ongoing digital skills development and training programs to enable employees to adapt to new technologies. Reskilling and career development programs help employees maintain their employability and reduce concerns about both job and occupation insecurity by increasing their confidence in their ability to adapt to technology-related workplace requirements.

The role of managers is particularly important, as it is their responsibility to pay attention not only to work results but also to the difficulties or concerns employees face, and to provide the necessary assistance or ensure it is provided. Managers should actively monitor employees' technology-related workloads, provide guidance and resources when challenges arise, and encourage open discussions about coping with technology-related stress. Communicating with subordinates about organizational changes, job security, and the anticipated impact of new technologies can help reduce uncertainty and alleviate feelings of job insecurity. It is also valuable to encourage employees to maintain a positive psychological climate and to foster collaborative, mutually supportive relationships among colleagues.

Finally, the results suggest that public-sector workers and managerial employees in both sectors experience greater technological overload than other groups of workers. Therefore, these groups may need targeted support measures, including specialized digital training, improved workload management practices, better access to technical support, and communication about technological changes and their impact on job roles and career prospects.

## 8 Conclusion

The business environment and technological advancements require organizations to continually strive for greater operational efficiency and to respond quickly and effectively to the dynamic business landscape, which is why they are increasingly implementing advanced information and communication technologies.

Technological progress implies that digital technologies provide greater communication opportunities, open up a wide world of information, and create tools that help work effectively. On the other hand, techno-overload is a technology-related job demand closely associated with workplace technostress (Tarafdar et al., 2007).

The results of our study, conducted with a sample of Lithuanian employees, revealed that techno-overload was positively associated with employee outcomes, including perceptions of job insecurity, intention to leave the organization, and occupation insecurity. Job insecurity also serves as a mediator in the relationships between techno-overload and both turnover intention and occupation insecurity. Employees who report higher techno-overload tend to fear possible dismissal, exhibit a stronger intention to leave the organization, and feel less secure in the labor market because their profession may change or become obsolete due to technological advancements. Projects of organizational interventions aimed at strengthening staff stability, adaptability, and resilience in the context of digital advancements and decreasing negative employee reactions to technological innovations should be comprehensive, encompassing not only techno-overload regulation but also the improvement of employees' professional competencies, creation of an optimal work environment, and implementing measures supporting fear balance in employer – employee relations.

## Data Availability

The raw data supporting the conclusions of this article will be made available by the authors, without undue reservation.
